# Lactase Persistence, Milk Intake, and Adult Acne: A Mendelian Randomization Study of 20,416 Danish Adults

**DOI:** 10.3390/nu10081041

**Published:** 2018-08-08

**Authors:** Christian R. Juhl, Helle K. M. Bergholdt, Iben M. Miller, Gregor B. E. Jemec, Jørgen K. Kanters, Christina Ellervik

**Affiliations:** 1 Department of Biomedical Sciences, Faculty of Health and Medical Sciences, University of Copenhagen, 2100 Copenhagen, Denmark; christian.r.juhl@gmail.com; 2 Department of Production, Research, and Innovation, Region Zealand, 4180 Sorø, Denmark; hellebergholdt@hotmail.com; 3 Department of Dermatology, Zealand University Hospital, 4000 Roskilde, Denmark; miller@dadlnet.dk (I.M.M.); gbj@regionsjaelland.dk (G.B.E.J.); 4 Department of Clinical Medicine, Faculty of Health and Medical Sciences, University of Copenhagen, 2100 Copenhagen, Denmark; 5 Department of Laboratory Medicine, Boston Children’s Hospital, 300 Longwood Avenue, Boston, MA 02115, USA; 6 Department of Pathology, Harvard Medical School, Boston, MA 02115, USA

**Keywords:** acne, acne vulgaris, milk, dairy, diet, Mendelian randomization, adults

## Abstract

Whether there is a causal relationship between milk intake and acne is unknown. We tested the hypothesis that genetically determined milk intake is associated with acne in adults using a Mendelian randomization design. *LCT-13910 C/T* (rs4988235) is associated with lactase persistence (*TT/TC*) in Northern Europeans. We investigated the association between milk intake, *LCT-13910 C/T* (rs4988235), and acne in 20,416 adults (age-range: 20–96) from The Danish General Suburban Population Study (GESUS). The adjusted observational odds ratio for acne in any milk intake vs. no milk intake was 0.93(95% confidence interval: 0.48–1.78) in females and 0.49(0.22–1.08) in males aged 20–39 years, and 1.15(95% confidence interval: 0.66–1.99) in females and 1.02(0.61–1.72) in males above 40 years. The unadjusted odds ratio for acne in TT+TC vs. CC was 0.84(0.43–1.62) in the age group 20–39 years, and 0.99(0.52–1.88) above 40 years. We did not find any observational or genetic association between milk intake and acne in our population of adults.

## 1. Introduction

Acne is a common chronic inflammatory skin disease, which is almost universal in adolescence, with rates up to 85% [1,2,3,4]. After adolescence the prevalence decreases but a significant number of patients are affected by persistent acne or develop new-onset adult acne [5]. Acne is overall characterized by open comedones, papules, pustules, and nodules [6], but the clinical appearance varies by age and lifestyle [5,7,8].

The genetic architecture of acne vulgaris is complex and multiple susceptible loci have been identified reflecting the multifactorial pathogenesis of acne involving the innate immune system, inflammation, modified lipogenesis, and androgens [9,10]. But there is likely also an environmental component in the development of acne vulgaris. Several observational studies have investigated the association of milk intake with acne in children, adolescents, and young adults [11,12,13,14,15,16]. However, no previous observational study has been performed in all adult ages.

In individuals of Northern European descent, the genetic variant *LCT-13910 C/T* (rs4988235) located 13,910 base pairs upstream of the lactase (*LCT*) gene on chromosome 2q21-22, within intron 13 of the adjacent *MCM6* gene, shows complete correlation with lactase persistence/non-persistence [17]. The T-allele is the lactase-persistent allele, whereas the C allele is the lactase non-persistent allele. The inheritance is autosomal recessive manner such that individuals homozygous for CC are unable to digest lactose, whereas individuals with TC or TT are able to digest lactose.

To investigate whether there is a causal relationship between milk intake and acne, large long-term randomized trials would be needed, but these are costly and it is difficult to uphold the randomization over time. Instead the epidemiological Mendelian randomization (MR) design offers a feasible alternative [18]. The underlying principle in the MR design is that genetic variants are randomly assorted during gamete formation, which is similar to the random assignment of patients to placebo or active treatment in a clinical intervention trial. In the MR design, confounders are, therefore, balanced across the genotypes, and the genotypes will serve as a proxy for lifelong exposure.

In this study, we used the Mendelian randomization design to investigate the long-term effect of milk intake on acne using the lactase persistent (TT + TC)/non-persistent(CC) *LCT-13910* C/T genotype in 20,416 adult individuals from The Danish General Population study (GESUS).

## 2. Materials and Methods

### 2.1. Participants

The cross-sectional population study The Danish General Population Study (GESUS) was conducted from January 2010 to October 2013 in Naestved Municipality, Denmark [19]. Criteria for invitation were age 20+, Danish citizenship and Danish Civil Registration number (CPR number).

All persons aged 30+ were invited and in the age group 20–30 years only a random 25% selection were invited. 21,205 adults were enrolled, with an overall participation rate of 43%.

In this present study, 20,850 persons was included of whom 98.9% were of Danish descent and the rest other Scandinavian or European descent. Individuals with missing values for acne diagnosis (*n* = 53) or *LCT-13910 C/T* genotyping (*n* = 381) were excluded, resulting in inclusion of 20,416 people.

A prerequisite for attending the health examination was a completed self-reported paper-questionnaire about demographic information, medical history, smoking, skin-condition, and food intake, among others. The health examination was performed by trained health professionals and took place at the department of clinical biochemistry at Naestved University Hospital, Denmark. Body mass index (BMI) was calculated as kg/m^2^. Details about the study design of GESUS have been described elsewhere [19].

Written informed consent was obtained from all participants. The study conforms to the principles of the Declaration of Helsinki and is approved by the institutional review board, the ethical committee of Region Zealand (SJ-113, SJ-114, SJ-191) and the Danish Data Protection Agency.

### 2.2. Milk Intake

Intake of milk was reported in the questionnaire as: “How much milk do you averagely consume per week?” and the possible answers were glasses of whole milk (3.5% fat), semi-skimmed milk (0.5–1.5% fat), skimmed milk (0.1–0.3% fat), butter milk, and lactose free milk. A blank response in one variable was set as no intake, when any of the other variables were filled. Extreme values were confirmed/deferred by contacting the participants by phone [19]. Milk intake was divided into categorical variables, based on glasses of milk per day. Dichotomized variables for types of milk intake were performed to compare no milk intake with intake of low-fat (0.1–0.3%) and high-fat milk (0.5–3.5%).

### 2.3. Acne Diagnosis

The acne diagnosis was based on the self-reported questionnaire validated by Dalgard et al. [20] The questions in the questionnaire were as follows: “During the last week, have you had any of the following complaints?” one of the complaints was pimples and the possible answers were: No (0); Yes, a little (1); Yes, quite a lot (2) or Yes, very much (3). The criteria used for the diagnosis of acne was answering “Yes, quite a lot (2)” or “Yes, very much (3)”. The validation study by Dalgard et al. showed a high specificity (96%) for a non-healthcare seeking population, but a low sensitivity (<50%) [20].

### 2.4. Genotyping

Every participant in the GESUS study was genotyped for *LCT-13910 C/T* variant (rs4988235). The genotyping was done by KASPar allelic discrimination (LGC Genomics) with a call rate of 99% [21]. The genotype *LCT-13910* TC and TT are lactase persistent and CC lactase non-persistent. The *LCT-13910* C/T genotype distribution, in the GESUS population, was in Hardy-Weinberg equilibrium (Supplementary Table S1).

### 2.5. Statistical Analyses

Statistical analyses were performed in SAS Enterprise Guide 7.1 (SAS institute Inc., Cary, NS, USA). The descriptive statistics of all the continuous variables showed normal distributions, except for milk intake. Transformation of the variable was performed, but this did not improve normal distribution substantially. Milk intake was instead categorized into quantiles (0, 1–3, 4–7, 8–14, >14 glasses/week) and dichotomized variables were performed for total milk, low-fat milk and high-fat milk. (any vs. none). Chi-square test and analysis of variance (ANOVA) was used to test relationships for categorical variables and differences in means. A *p*-value < 0.05 was considered significant. The Mendelian randomization design consisted of three analyses: First, logistic regressions were performed to test the observational associations of milk intake and acne. The logistic regressions were stratified by age, based on an empirical data description of milk intake and acne diagnosis (Supplementary Table S2). This resulted in the two age groups; 20–39 years and 40+ years. Furthermore, stratification by gender was performed, because of interaction with milk intake (*p =* 0.003) The logistic regressions were performed both unadjusted and adjusted for age, body mass index (BMI) and smoking, as BMI and smoking are related to acne in the literature [22,23]. Second, the median milk intake was studied for the *LCT-13910* C/T genotypes and the difference was tested with the Kruskal-Wallis test. Finally, logistic regressions were performed for the *LCT-13910* C/T genotypes and acne. An interaction test was performed for milk intake (no/yes) and lactase genotype in an additive (*TT; TC; CC*) and dominant (*TT/TC; CC*) model in both age groups.

### 2.6. Meta-Analysis of Acne in Adults

The aim was to meta-analyze the association between milk intake and acne in adults.

The search was performed on 11 December 2017 and included all studies up until that date. Studies were identified in the PubMed database using the search terms: (“Dairy products”[Mesh] OR “dairy”[All Fields] OR “milk”[Mesh] OR “milk”[All Fields] OR yogurt[All Fields] OR cheese[All Fields] OR lifestyle[All Fields]) AND (“Acne Vulgaris”[Mesh] OR “Acne”[All Fields]). We identified 241 records. Inclusion criteria was mean age ≥ 30 years, case (acne) and control (non-acne) groups, and information on odds ratio (95%CI) or raw numbers to calculate the odds ratio. Milk intake was defined as binary (yes, no), low-fat (yes/no), or high-fat (whole) milk (yes/no). We identified two studies of milk intake and adult acne [24,25], but only study met the inclusion criteria for case-control group design [25] which we meta-analyzed with our own results. We calculated pooled fixed and random effects odds ratios.

## 3. Results

### 3.1. Baseline Characteristics

Baseline characteristics of the 20,416 participants by acne, age group and *LCT-13910* genotype are presented in Table 1 and Supplementary Table S2. The acne group consisted of 303 participants, which were significantly younger, had a higher percentage of smokers, as well as a higher mean milk intake. The group aged 20–39 years had significantly fewer men, lower BMI, higher prevalence of acne, and a higher milk intake, compared to the group aged 40+. The lactase genotype groups were significantly different, with a higher BMI and milk intake in the lactase persistent TC/TT genotypes, compared to the lactase non-persistent genotype (CC).

### 3.2. Milk Intake and Acne

The adjusted observational odds ratio for acne in individuals with any milk intake vs. no milk intake was 0.93(95% confidence interval: 0.48–1.78) in females and 0.49(0.22–1.08) in males aged 20–39 years, and 1.15(95% confidence interval: 0.66–1.99) in females and 1.02(0.61–1.72) in males above 40 years (Table 2 and Table 3). Results were similar for unadjusted analyses.

### 3.3. Lactase Genotype and Milk Intake

In the age group 20–39 years, the median milk intake (Table 4) for the lactase persistent genotypes was 10 glasses/week (inter-quantile range (IQR) [4:16]) for TT and 10 glasses/week [3:16] for TC compared to 7 glasses/week [2:14] for the non-persistent lactase genotype CC (*p =* 6.32 × 10^−4^). In the age group 40+ (Table 4), results were similar but attenuated with a median milk intake of 5 glasses/week [IQR: 0:10] for TT and 6 glasses/week [0:14] for TC compared to 3 glasses/week [0:14] for CC (*p =* 3.78 × 10^−12^).

### 3.4. Lactase Genotype and Acne

The unadjusted odds ratio for acne in individuals with the lactase persistent genotypes TC/TT vs. the lactase non-persistent genotype CC was 0.84 (0.43:1.62) in the age group 20–39 years, and 0.99 (0.52–1.88) above 40 years. (Table 5). No interactions were found for the lactase genotype and milk (no/yes) for both the additive and dominant model, in both age groups.

### 3.5. Meta-Analysis of Milk Intake and Adult Acne

Combining all age groups in our own study with the study by Landro [25], the pooled fixed effects odds ratios for acne was 1.04(0.79–1.37) for any milk intake (yes/no), 1.05(0.70–1.58) for whole milk, and 1.02(0.78–1.34) for low-fat milk intake (Table 6).

## 4. Discussion

Among 20,419 adults from the Danish general population we found no association between milk intake and acne, observationally or genetically using the lactase persistent/non-persistent *LCT-13910 C/T* genotype in a Mendelian randomization design.

In the Mendelian randomization design the genetic variant is used as a proxy for the long-term differences in milk intake, thereby largely avoiding confounding and reverse causation, which can blur or distort the underlying true association in observational studies [18]. The *LCT-13910* fulfilled the requirements for using the Mendelian randomization design, as the variant is linked to the intermediate phenotype (milk intake) in a biologically explainable way [17], there is no known pleiotropic effects of the variant, and the variant was not associated with confounders. The Mendelian randomization design mimics a randomized clinical trial and takes advantage of the random assortment of alleles at conception which ensures random distribution of confounding factors thereby circumventing reverse causation and most confounding. Thus, the Mendelian randomization design provides an estimate of the long-term effect of milk intake on acne.

We showed that both the milk consumption and the acne diagnosis declined with age, and that fewer people drank milk as age increased. Nevertheless, we found a crude prevalence of self-reported acne of 6%. We also investigated milk intake and acne among adults by combining our current study with findings from Landro , but the associations were still null for any milk intake, whole milk, and low-fat milk. In contrast, observational studies of mostly childhood and adolescent acne have debated, but largely favored, the association between milk intake and acne [11,12,13,16,26,27,28,29]. These studies were heterogeneous with respect to geographical location, cultural dairy influence, gender, sample size, reporting of dairy frequency and type, and the ascertainment of participants (dermatology clinics, general population study, online questionnaire). Some of the studies did not run adjusted analyses [16,26,27], thus confounding may account for the associations. However, reasons for the discrepant findings between our study and the study by Landro [25] in adults versus the studies in adolescent acne [11,12,13,16,26,27,28,29] could also be related to different pathogenesis and the appearance of acne in different age groups. Milk intake increases levels of insulin-like growth factor-1 (IGF1), which is hypothesized to be a central link between milk intake and stimulation of the sebaceous gland [30]. Thus, as people age and drink less milk, they may be less exposed to IGF1 and, therefore, also less prone to the development of acne. The appearance of acne varies by age, such that acne precox and acne tarda are mostly variants with predominant inflammatory papules, pustules and nodules at the lower face half [5,31], while adolescent acne is mostly characterized by comedoes and papules/pustules at the entire face (chest and back) [7]. In contrast, acne in adult smokers is characterized by comedoes and scars [8] and acne fulminans exhibits a severe clinical picture [32]. Even though the acne diagnosis was questionnaire based as in many previous observational studies [16,28], it has been validated in a comparable setting with a high specificity but with a sensitivity of only 50% [20]. However, we did not have information on anatomical acne location or longer period of acne appearance. Additionally, our study spanned four years including all seasons, thereby compensating for seasonal variation in acne appearance [33].

The strength of our study is the homogenous population of largely Danish descent or other Scandinavian descent making confounding from population substructure less likely. Similarly, the rs4988235 variant is the most common lactase persistent variant in populations of Northern European descent, despite the many other lactase persistence haplotypes [34]. A limitation of our study included the low prevalence of lactase non-persistence (6%) and the fact that some of these individuals drank milk, which could offset and conceal a true association. Milk consumption was based on self-reported questionnaire data, with possible recall bias.

## 5. Conclusions

In conclusion, in the Danish General Suburban Population Study (GESUS) of adults we did not find any observational or genetic association between milk intake and acne using the lactase persistent/non-persistent *LCT-13910* C/T genotype in a Mendelian randomization design.

## Figures and Tables

**Table 1:** Baseline characteristics for The Danish General Suburban Population study (GESUS) by acne, age groups, and *LCT-13910* genotype.

	All *n* = 20,416 100%	Acne *n* = 303 1.5%	Control *n* = 20,113 98.5%	*p*-Value *	Age 20–39 *n* = 2742 13.4%	Age > 40 *n* = 17,674 86.6%	*p*-Value *	*LCT*-13910 Genotype			*p*-Value **
								CC *n* = 1246 6.1%	TC *n* = 7377 36.1%	TT *n* = 11,793 57.8%	
**Acne,** ***n* (%)**	303 (1.5)	-	-	-	141 (5.1)	162 (0.9)	<0.0001	20 (1.6)	119 (1.6)	164 (1.4)	0.4523
**Age,** **mean (SD), years**	56.3 (13.6)	44.2 (13.2)	56.5 (13.5)	<0.0001	35.0 (4.0)	59.7 (11.3)	<0.0001	55.7 (13.2)	56.4 (13.6)	56.4 (13.6)	0.1892
**Age: 20–39,** ***n* (%)**	2742 (13.4)	141 (46.5)	2601 (12.9)	<0.0001	-	-	-	166 (6.1)	955 (34.8)	1621 (59.1)	0.2851
**Age: > 40,** ***n* (%)**	17674 (86.6)	162 (53.5)	17512 (87.1)	<0.0001	-	-	-	1080 (6.1)	6422 (36.3)	10172 (57.6)	
**Men,** ***n (%)***	9294 (45.5)	125 (41.3)	9169 (44.9)	0.1327	1193 (43.5)	8101 (45.8)	0.0228	540 (43.3)	3332 (45.2)	5422 (46.0)	0.1522
**Body Mass Index,** **mean (SD), kg/m^2^**	26.7 (4.7)	26.9 (5.6)	26.7 (4.7)	0.4549	25.9 (4.9)	26.9 (4.6)	<0.0001	26.5 (4.7)	26.6 (4.6)	26.8 (4.7)	0.0290
**Current Smoker,** ***n* (%)**	3632 (17.8)	75 (24.8)	3557 (17.7)	0.0014	490 (17.9)	3142 (17.8)	0.9060	233 (18.7)	1318 (17.9)	2081 (17.7)	0.6371
**Milk Intake,** **mean (SD), glasses/week**	8.1 (9.1)	10.2 (14.3)	8.1 (9.0)	<0.0001	11.0 (9.9)	7.7 (8.9)	<0.0001	6.1 (7.1)	8.2 (9.1)	8.3 (9.4)	<0.0001

* The chi-square test or the analysis of variance (ANOVA) test is used to calculate the *p*-value. ** Rao-Scott modified chi-square test is used for categorized variables and ANOVA for continuous variables. *LCT-13910* genotype (rs4988235): CC = the lactase non-persistent genotype, TC/TT = the lactase persistent genotype.

**Table 2:** Odds ratio for acne by milk intake in the age group 20–39 years.

Milk Intake (glasses/week)	Female							Male				
	*n* Total	*n* Acne	Unadjusted		Adjusted *		*n* Total	*n* Acne	Unadjusted		Adjusted *	
			OR [95% CI]	*p*	OR [95% CI]	*p*			OR [95% CI]	*p*	OR [95% CI]	*p*
0	172	11	1.00	-	1.00	-	122	8	1.00	-	1.00	-
1–3	274	19	1.09 [0.51:2.35]	0.82	0.98 [0.45:2.13]	0.96	147	4	0.40 [0.12:1.36]	0.14	0.43 [0.13:1.47]	0.18
4–7	375	25	1.05 [0.50:2.18]	0.91	0.98 [0.47:2.06]	0.96	186	11	0.90 [0.35:2.29]	0.82	0.93 [0.36:2.41]	0.89
8–14	397	25	0.98 [0.47:2.05]	0.96	0.90 [0.43:1.90]	0.79	322	7	0.32 [0.11:0.89]	0.03	0.36 [0.13:1.02]	0.06
>14	331	20	0.94 [0.44:2.01]	0.88	0.85 [0.39:1.83]	0.67	416	11	0.39 [0.15:0.98]	0.05	0.40 [0.16:1.02]	0.05
Any	1377	89	1.01 [0.53:1.93]	0.97	0.93 [0.48:1.78]	0.82	1071	33	0.45 [0.20:1.00]	0.05	0.49 [0.22:1.08]	0.08
Low-fat **	1348	85	0.83 [0.47:1.48]	0.53	0.84 [0.47:1.52]	0.57	1031	31	0.47 [0.23:0.98]	0.04	0.49 [0.23:1.03]	0.06
High-fat ***	58	7	2.06 [0.91:4.67]	0.08	1.42 [0.55:3.69]	0.47	82	5	1.94 [0.74:5.08]	0.18	2.01 [0.75:5.38]	0.17

* Adjusted for age, smoking and body mass index, ** Low-fat (0.1–0.3%), *** High-fat (0.5–3.5%). *p*-values are calculated as analysis of maximum likelihood estimates.

**Table 3:** Odds ratio for acne by milk intake for in the age group ≥40 years.

Milk Intake (glasses/week)	Female							Male				
	*n* Total	*n* Acne	Unadjusted		Adjusted *		*n* Total	*n* Acne	Unadjusted		Adjusted *	
			OR [95% CI]	*p*	OR [95% CI]	*p*			OR [95% CI]	*p*	OR [95% CI]	*p*
0	2802	17	1.00	-	1.00	-	2099	20	1.00	-	1.00	-
1–3	1564	16	1.69 [0.85:3.36]	0.13	1.30 [0.65:2.59]	0.46	1099	10	0.95 [0.45:2.05]	0.90	0.91 [0.42:1.96]	0.80
4–7	2129	22	1.71 [0.91:3.23]	0.10	1.40 [0.74:2.65]	0.31	1592	13	0.86 [0.42:1.73]	0.66	0.82 [0.40:1.68]	0.59
8–14	1816	11	1.00 [0.47:2.14]	1.00	0.76 [0.35:1.63]	0.48	1682	23	1.44 [0.79:2.63]	0.23	1.31 [0.71:2.43]	0.39
>14	1262	12	1.57 [0.75:3.30]	0.23	1.13 [0.53:2.40]	0.75	1629	18	1.16 [0.61:2.20]	0.65	0.99 [0.51:1.91]	0.97
Any	6771	61	1.49 [0.87:2.55]	0.15	1.15 [0.66:1.99]	0.62	6002	64	1.12 [0.68:1.86]	0.66	1.02 [0.61:1.72]	0.94
Low-fat **	6324	58	1.49 [0.90:2.49]	0.12	1.14 [0.68:1.91]	0.63	5268	57	1.14 [0.72:1.80]	0.59	0.98 [0.61:1.58]	0.94
High-fat ***	590	4	0.82 [0.30:2.26]	0.70	0.97 [0.35:2.68]	0.95	901	10	1.08 [0.56:2.10]	0.82	1.25 [0.64:2.46]	0.51

* Adjusted for age, smoking and body mass index, ** Low-fat (0.1–0.3%), *** High-fat (0.5–3.5%). *p*-values are calculated as analysis of maximum likelihood estimates.

**Table 4:** Differences in milk intake, glasses per week, by the *LCT-13910 C/T* genotype.

*LCT*-13910 Genotype	Lactase	*n*	%	Median	IQR	Kruskal-Wallis Test	
**Age Group 20–39 Years**							
CC	Non-persistent	166	6	7	[2:14]	Chi-Square	14.73
TC	Persistent	955	35	10	[3:16]	DF	2
TT	Persistent	1621	59	10	[4:16]	P	6.32 × 10^−4^
**Age Group ≥ 40 Years**							
CC	Non-persistent	1080	6	3	[0:14]	Chi-Square	52.83
TC	Persistent	6422	36	6	[0:14]	DF	2
TT	Persistent	10,172	58	5	[0:10]	P	3.38 × 10^−12^

IQR: Inter-quartile range.

**Table 5:** Odds ratio for acne by *LCT-13910 C/T* genotypes.

Median Milk Intake [IQR]					
*LCT-13910 C/T* Genotype	Glasses/Week	*n* Total	*n* Acne	OR [95% CI]	*p*-Value
**Age Group 20–39 Years**					
CC	7 [2:14]	166	10	1	-
TC	10 [3:16]	955	50	0.86 [0.43:1.74]	0.68
TT	10 [4:16]	1621	81	0.82 [0.42:1.62]	0.57
TC/TT	10 [3:16]	2576	131	0.84 [0.43:1.62]	0.60
*LCT* gene (additive model) × milk (no/yes) interaction test *LCT* gene (dominant model) × milk (no/yes) interaction test					0.36
					0.15
**Age Group ≥ 40 Years**					
CC	3 [0:10]	1080	10	1	-
TC	6 [0:14]	6422	69	1.16 [0.60:2.26]	0.67
TT	5 [0:14]	10,172	83	0.88 [0.46:1.70]	0.70
TC/TT	5 [0:14]	16,594	152	0.99 [0.52:1.88]	0.97
*LCT* gene (additive model) × milk (no/yes) interaction test *LCT* gene (dominant model) × milk (no/yes) interaction test					0.32
					0.37

**Table 6:** Meta-analysis of milk intake and acne in adults.

Dairy	Author	Year	OR	Low 95% CI	Upper 95% CI	*p*-Value
Any intake	Di Landro [25]	2016	0.88	0.61	1.28	0.52
Any intake	Juhl	Current	1.27	0.84	1.92	0.26
*Fixed effects pooled odds ratio*			1.04	0.79	1.37	0.78
*Random effects pooled odds ratio*			1.05	0.74	1.49	0.80
Whole milk	Di Landro [25]	2016	0.84	0.49	1.44	0.53
Whole milk	Juhl	Current	1.43	0.76	2.70	0.27
*Fixed effects pooled odds ratio*			1.05	0.70	1.58	0.82
*Random effects pooled odds ratio*			1.07	0.64	1.79	0.81
Low-fat milk	Di Landro [25]	2016	0.90	0.61	1.32	0.59
Low-fat milk	Juhl	Current	1.15	0.79	1.68	0.47
*Fixed effects pooled odds ratio*			1.02	0.78	1.34	0.89
*Random effects pooled odds ratio*			1.02	0.78	1.34	0.89

## Supplementary Materials

The following are available online at http://www.mdpi.com/2072-6643/10/8/1041/s1, Table S1: Hardy-Weinberg equilibrium test; Table S2: Characteristics by age groups.
