# Supplementary material for: Lactase Persistence, Milk Intake, and Adult Acne: A Mendelian Randomization Study of 20,416 Danish Adults
Source: Nutrients. 2018 Aug 8;10(8):1041. doi: 10.3390/nu10081041 (PMC6115808; doi:10.3390/nu10081041)
Supplement: Supplementary file 1 [file nutrients-10-01041-s001.pdf]

Supplementary data

Supplementary Table 1: Hardy-Weinberg equilibrium test

LCT-13910 C/T genotypes	Number observed	Number expected	Hardy Weinberg disequilibrium coefficient	x^2	p-value
Age 20-39					
CC	166	151	0.0055	2.54	0.11
TC	955	985			
TT	1621	1606			
**Age group ≥40 years**					
CC	1080	1042	0.0022	2.44	0.12
TC	6422	6498			
TT	10172	10134			

Supplementary Table 2: Characteristics by age groups.

	**Age groups:**							
	**20-29** (n=293)	**30-39** (n=2450)	**40-49** (n=4324)	**50-59** (n=4618)	**60-69** (n=5382)	**70-79** (n=2642)	**80-89** (n=678)	**90-99** (n=36)
Male, *N(%)*	129 (44.18)	1064 (43.23)	1886 (43.62)	2057 (44.54)	2553 (47.44)	1258 (47.62)	331 (49.26)	16 (44.44)
Acne, [2+3] *N(%)*	24 (8.22)	117 (4.78)	83 (1.92)	40 (0.87)	20 (0.37)	14 (0.53)	5 (0.74)	0 (0.00)
Any milk intake, *N(%)*	259 (88.70)	2189 (89.35)	3573 (82.63)	3304 (71.55)	3618 (67.22)	1772 (67.07)	480 (71.43)	26 (72.22)
Current smoker, *N(%)*	69 (23.63)	421 (17.18)	822 (19.01)	931 (20.16)	967 (17.97)	356 (13.47)	64 (9.52)	2 (5.56)
BMI, *mean(SD)*	25.47 (4.91)	25.96 (4.87)	26.46 (4.74)	26.84 (4.66)	27.11 (4.64)	27.17 (4.56)	26.23 (4.11)	25.38 (3.73)
**"Pimples the last week?"** *N(%)*								
Did not answer	2 (0.68)	25 (1.02)	51 (1.18)	132 (2.86)	228 (4.24)	184 (6.96)	82 (12.20)	3 (8.33)
[0] No	87 (29.76)	1274 (52.00)	3038 (70.26)	3849 (83.35)	4874 (90.56)	2372 (89.78)	568 (84.52)	33 (91.67)
[1] Yes, a little	179 (61.30)	1034 (42.20)	1152 (26.64)	597 (12.93)	260 (4.83)	72 (2.73)	17 (2.53)	0 (0.00)
[2] Yes, quite a lot	20 (6.85)	97 (3.96)	66 (1.53)	33 (0.71)	15 (0.28)	9 (0.34)	4 (0.60)	0 (0.00)
[3] Yes, very much	4 (1.37)	20 (0.82)	17(0.39)	7 (0.15)	5 (0.09)	5 (0.19)	1 (0.15)	0 (0.00)
